# Composition, Diversity and Functional Analysis of the Modern Microbiome of the Middle Triassic Cava Superiore Beds (Monte San Giorgio, Switzerland)

**DOI:** 10.1038/s41598-019-55955-5

**Published:** 2019-12-31

**Authors:** Sania Arif, Joachim Reitner, Michael Hoppert

**Affiliations:** 1 Institute of Microbiology & Genetics, Department of General Microbiology, Georg-August-Universität Göttingen, Göttingen, Germany; 2 Geosciences Centre, Department of Geobiology, Georg-August-Universität Göttingen, Göttingen, Germany

**Keywords:** Bacterial techniques and applications, Metagenomics, Environmental impact

## Abstract

Organic-rich laminated shales and limestones from the Monte San Giorgio (Lugano Prealps, Switzerland) are known as famous fossil lagerstätten for excellently preserved fossils from the Middle Triassic Period. The various bituminous shales from Monte San Giorgio are thermally immature and rich in diverse organic compounds, which provide unique substrates for active soil microbial communities. We selected the Cava superior beds of the Acqua del Ghiffo site for this study. To investigate its microbial structure and diversity, contig assembly, Operational Taxonomic Units (OTUs) clustering, and rarefaction analysis were performed for bacterial 16S rDNA preparations from bituminous and non-bituminous limestone strata with the MetaAmp pipeline. Principal coordinates analysis shows that the microbial communities from the bituminous strata differ significantly from limestone samples (*P* < *0.05* Unifrac weighted). Moreover, metagenomic tools could also be used effectively to analyze the microbial communities shift during enrichment in specific growth media. In the nutrient-rich media, one or few taxa, mainly *Proteobacteria* and *Firmicutes*, were enriched which led to the drastic diversity loss while oligotrophic media could enrich many taxa simultaneously and sustain the richness and diversity of the inoculum. Piphillin, METAGENassist and MicrobiomeAnalyst pipeline also predicted that the Monte San Giorgio bituminous shales and oligotrophic enriched microbiomes degrade complex polycyclic aromatic hydrocarbons.

## Introduction

Monte San Giorgio, located near Lake Lugano in Canton Ticino, Switzerland, is listed as a UNESCO World Heritage site because of its best preserved paleontological record from the Middle Triassic Period (approx. 245–230 million years ago)^1,2^. Many abundant and exceptionally detailed fossils of reptiles, fish, bivalves, ammonites, echinoderms and crustaceans, insects, and plants have been reported^3^. During the later Middle Triassic, the region around Monte San Giorgio formed restricted basins that were largely isolated from the open ocean with an offshore reef^4–6^. Being close to land, this basin included both diverse marine and terrestrial life under nearly perfect stagnant, euxinic, or even anoxic conditions. Successive phases of marine transgression and regression created different environments that allowed for the deposition of fine-grained, mostly laminated mudstones, limestones, and dolomites with varying content of organic material (up to 40%)^7–9^. The Middle Triassic sequence consists of approximately 1,000 metres of reef limestones, dolomites, and bituminous shales which formed in marine conditions at the southern margin of the Triassic ‘Tethys’ Ocean. At least five distinct fossiliferous formations within the Middle Triassic Lower Meride limestone are the Grenzbitumenzone, Cava Inferior, Cava Superior, Cassina Beds and Kalkschieferzone, each yielding different vertebrate assemblages^5,10,11^ and consisting of finely laminated limestones and marls with intercalated volcanic ash layers of andesitic and rhyolitic composition.

Many scientific reports document the fossils of Monte San Giorgio but no studies have been conducted to evaluate its extant geomicrobiology, though bituminous shales may provide substrates suitable for microbial growth. These shales provide rather an immature bitumen and partly kerogen, consisting of diverse organic compounds, among them hopanes, hopenes, and methylhopanoids^12^ and their metagenomic biodiversity analysis of the non-culturable microbiome is interesting from a microbiological, geochemical as well as a biotechnological point of view. The aim of this study was to explore the microbial structure and functional diversity of the Monte San Giorgio organic-rich shale and understand its contribution towards the carbon cycle through degradation of the associated complex hydrocarbons. The other objective was to investigate how the microbial growth and diversity can be influenced by different enrichment media and which hydrocarbon-degrading metabolic groups may be enriched from the environment. For this purpose, we examined the microbial communities of Monte San Giorgio organic-rich shale and limestone rocks samples via Illumina MiSeq sequencing, followed by microbial growth in enrichment media to elucidate the correlation of the microbial communities and enrichment effect of media. Paired-end sequencing was performed by Göttingen Genomics Laboratory after indexing of the V3-V4 PCR amplicons with the Nextera XT DNA library prep kit (Illumina, San Diego, Cal, USA). In addition, the geochemical and bioremediation metabolic potential of the microbiomes were predicted with the online available tools METAGENassist^13^ and Piphillin^14^.

## Results

### Metagenomics of Monte San Giorgio samples

**Figure 1:**
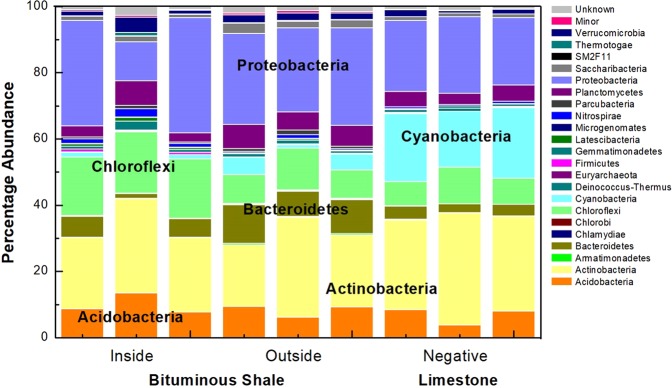
Relative abundance and taxonomic assignments of microbial flora at phylum level of inside and outside surfaces of the bituminous shale and limestone as the negative control in triplicates. Minor phyla having <0.02 relative abundance are represented as “Minor”.

A total of 4113 distinct OTUs were affiliated to 37 known phyla in the Monte San Giorgio samples. *Actinobacteria* (25.8% ± 4.6) and *Proteobacteria* (25.08% ± 6.5) were the most abundant phyla in all samples, followed by *Bacteroidetes* (5.9% ± 3.25), *Acidobacteria* (8.2% + 2.5), *Planctomycetes* (5.1% ± 1.6), *Verrucomicrobia* (3.6% ± 1.1), and *Saccharibacteria* (1.6% ± 0.65) (Fig. 1). *Cyanobacteria* was the most prevalent phylum in the limestone samples while *Chloroflexi* and *Latescibacteria* were abundant in the organic-rich shale samples. The analysis of variance (ANOVA) validated that the outside rock surface sample of the shale was prevalent in *Armatimonadetes*, *Saccharibacteria*, *SM2F11*, *Chlorobi*, *Bacteroidetes*, and *TM6* (*p* < 0.05). *Proteobacteria* subclass *Gammaproteobacteria* was relatively abundant in the inside surface of a freshly cleaved organic-rich shale sample (54%), whereas in limestone samples, *Alphaproteobacteria* (84%) were prevalent. More in-depth analysis at the order level with the hierarchical clustering and heatmap visualization illustrated the unique geomicrobiological fingerprint of each biospecimen (Fig. S1). The outside surface of the bituminous shale contained at least seven strictly aerobic or facultative orders (*Desulfurellales, Flavobacteriales, Rhizobiales, Xanthomonadales Sphingobacteriales, Sphingomonadales, and Caulobacteriales*) which are known for aerobic hydrocarbon metabolism^15^. In contrast, the organic-rich shale contained nine strictly anaerobic orders (*Thermotogales, Thermoplasmatales, Desulfobacterales, Chromatiales, Desulfuromonadales*, *Hydrogenophilales, Campylobacterales*, *Rhodocyclales, and Clostridiales*) exclusively.

These anaerobic orders may be potentially involved in the hydrocarbon metabolism along with the only three abundant aerobic orders (*Bacillales, Lactobacillales, and Enterobacteriales*)^15^. The limestone rocks were abundant in the orders *Rubrobacterales, Deinococcales, Rhodobacterales, Streptomycetales*, which have the ability to survive harsh environmental conditions such as high ionizing radiation^16–18^.

### Effect of enrichment media on the relative abundance of microbes

**Figure 2:**
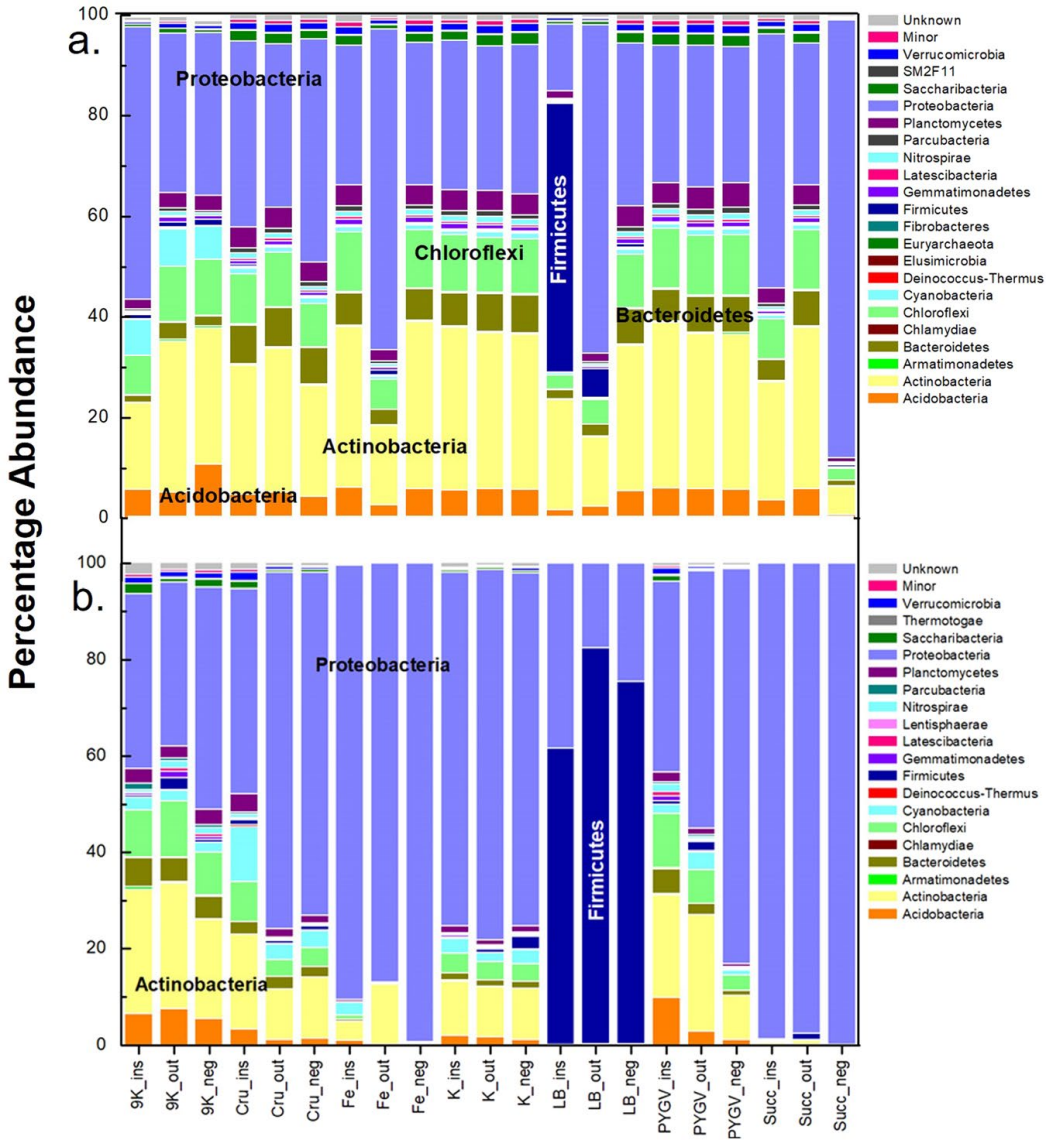
Relative abundance of microbial flora in the different enrichment media. (**a**) First and (**b**) third round of enrichments of the outside (out) and inside (ins) organic-rich shale and limestone negative control (neg) sample inoculums in 9 K (Silverman and Lundgren medium) Cru (Crude oil minimal media), K (Keller medium), LB (Luria-Bertani broth), PYGV (Peptone, Yeast extract, Glucose and vitamin mixture and mineral salts), Fe (Iron basal media), and Succ (Succinate minimal media).

After the first round of enrichment, *Proteobacteria*, *Bacteroidetes*, *Actinobacteria*, and *Chloroflexi* were found to be relatively abundant in almost every media. The selective effect of the culture media was not evident at this stage since the taxa of the natural rock environment were not displaced yet (Fig. 2a). After the final round of enrichment, the number of OTUs decreased from 5906 to 3296 which indicates the loss of diversity and microbial richness as the microbes most favorably adapted to the culture media were selected over others during the serial inoculum enrichment. *Firmicutes* was significantly enriched in the LB media, while *Proteobacteria* predominated in the succinate minimal and iron basal media. Interestingly, 9 K media, PYGV, and 1% crude oil minimal media were still rich in biodiversity and accommodated more diverse phyla and orders than other media (Fig. 2b). In the heatmap, the orders enriched in the nutrient-rich complex and oligotrophic media were not only distinguishable but also more abundant in the oligotrophic media (Fig. S2). The effect of each medium in enriching specific organisms was also observable, for instance, the LB media has enriched *Pseudomonadales*, and *Bacillales*, while the K media has enriched the soil/cave related orders *Rhizobiales, Ktedonobacterales, Acidobacteriales*, and *Armatimonadales*.

### Alpha diversity

**Figure 3:**
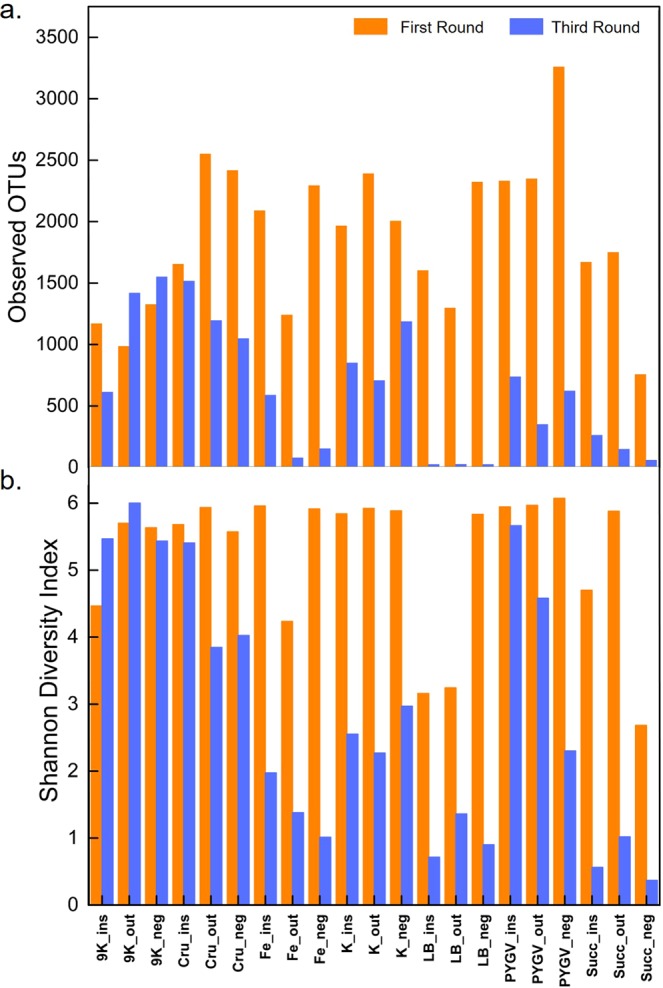
The number of observed OTUs (**a**) and Shannon diversity index (**b**) after the first and third round of enrichments. See Fig. 2 for abbreviations. Note that the nutrient-rich complex media have lost OTUs by more than 80%. The oligotrophic media are higher in alpha diversity than the nutrient-rich complex media.

Diversity indices provide important quantitative information about rarity and commonness of species which helps to understand community structure. The alpha diversity index Chao1 and abundance-based coverage estimators (ACE) indicated that richness of the samples was outside shale surface > inside shale surface > negative limestone control. The microbial species (OTUs) were more abundant on the outside surface of the shale sample than on inside shale surface and on limestone samples (Table S1). Following the serial inoculum enrichment, the subcultures drastically lost their alpha diversity especially when carbon/nitrogen sources were very rich, such as in LB complex media. The loss of OTUs also reduced both richness (Chao1 and ACE) and diversity (Shannon and Simpson) indexes, indicating that only a few adapted microbes were selected over others in the enrichment media (Fig. 3a). Nevertheless, the oligotrophic media provided more stringent conditions where the fast-growing bacteria did not utilize the carbon source to simply outgrow the rest; hence the species richness and diversity seem to be preserved (Fig. 3b).

### Beta diversity

**Figure 4:**
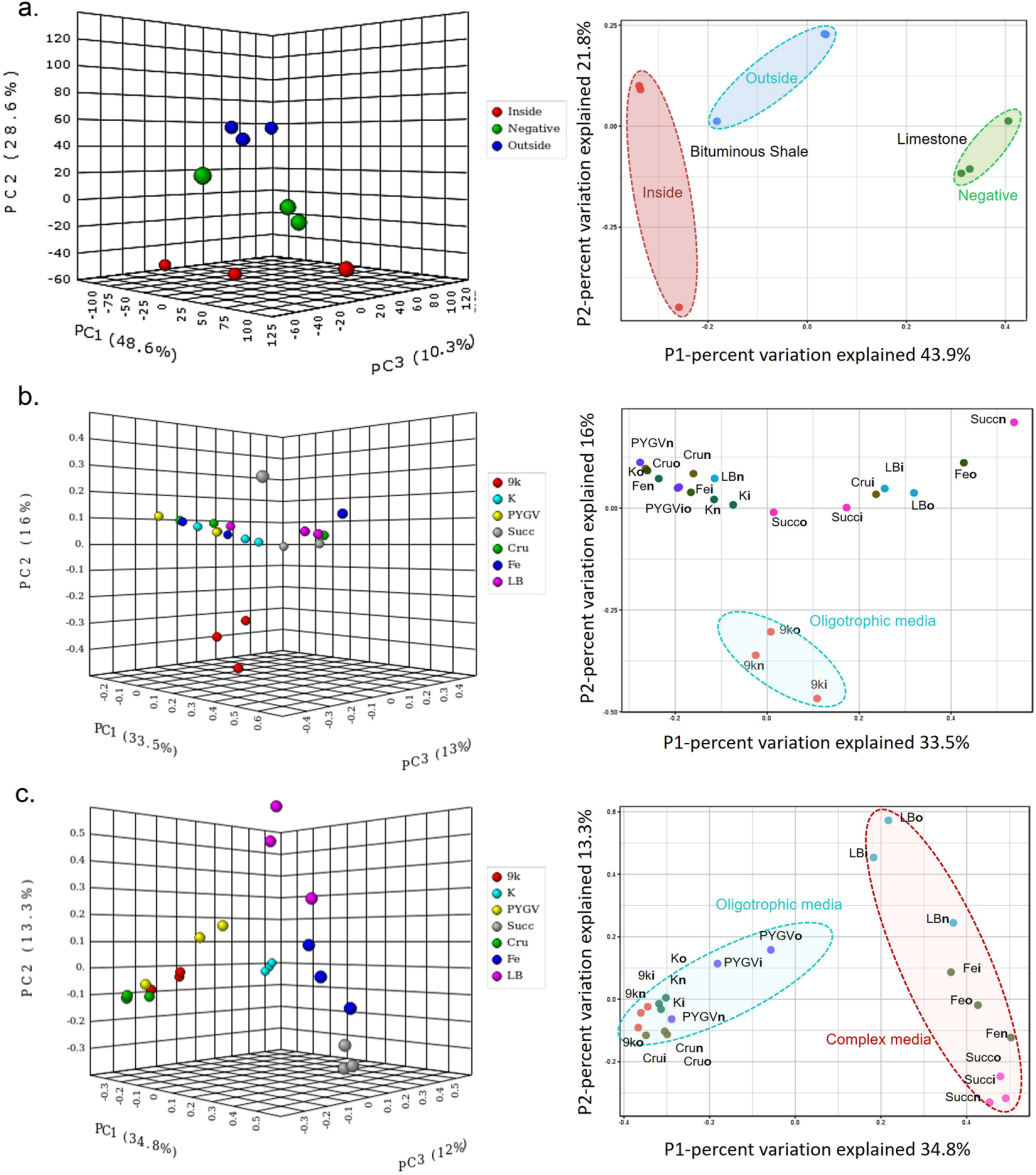
Principal Coordinates Analysis (PCoA) of the Monte San Giorgio samples and its enrichment cultures. PCoA of (**a**) inside, outside of bituminous shale and limestone rocks (negative), (**b**) enrichment cultures after the first round of incubation (**c**) third and final round of enrichments from inside, outside of bituminous shale and negative control: limestone rocks. See Fig. 2 for abbreviations of media; **i** (inside), **o** (outside) and **n** (negative control) are appended to an abbreviation.

Principal Coordinates Analysis (PCoA) is a multidimensional scaling method to explore and to visualize similarities or dissimilarities of complex metagenomic data into 2–3 dimensions. The PCoA of Monte San Giorgio samples showed that the microbial communities of inside, outside shale surface, and limestone samples were distinct to each other. The sample triplicates resemble each other more closely, though a considerable variance is obvious (Fig. 4a). This hypothesis was tested with the Unifrac weighted algorithm^19^. The significance between the inside and outside surfaces of the bituminous shale was <*0.0010*, same as the outside and limestone samples indicating that both populations are similar to each other but the organic-rich (inside) and limestone samples differ from each other (*p* = *0.0190*). The results were also validated with the analysis of molecular variance (AMOVA) nonparametric method^20^. AMOVA interprets whether genetic diversity within two populations is significantly different from their pooled populations or not (Table S2). It can be assumed that the different microorganisms from soil are being selected by these diverse environmental conditions as the soil-contaminated outside surface of shale’s microbiome shared similarity with both organic-rich bituminous shale and nutrient-starved limestone microbiomes.

### Effect of media and time in the enrichment process

The PCoA plots after the first round of incubation showed that 9 K media selected similar sets of microbes from the different samples, forming a separate cluster, distinct from all other media which promote growth of a set of further microbial communities, very similar to each other. Obviously, the effect of these other media in selecting different microbes was not substantial at this stage of the enrichment process (Fig. 4b). After the third round of serial subculturing, the effect of media in selecting the same set of organisms from different samples was profound, implying the same environmental conditions selected the same microbes over the passage of time. 9 K, K, crude oil, and, PYGV selected sets of microbes forming a cluster distinct from that of Fe basal media, succinate minimal, and LB media (Fig. 4c).

### Functional profile analysis

#### Taxonomic to phenotypic mapping

**Figure 5:**
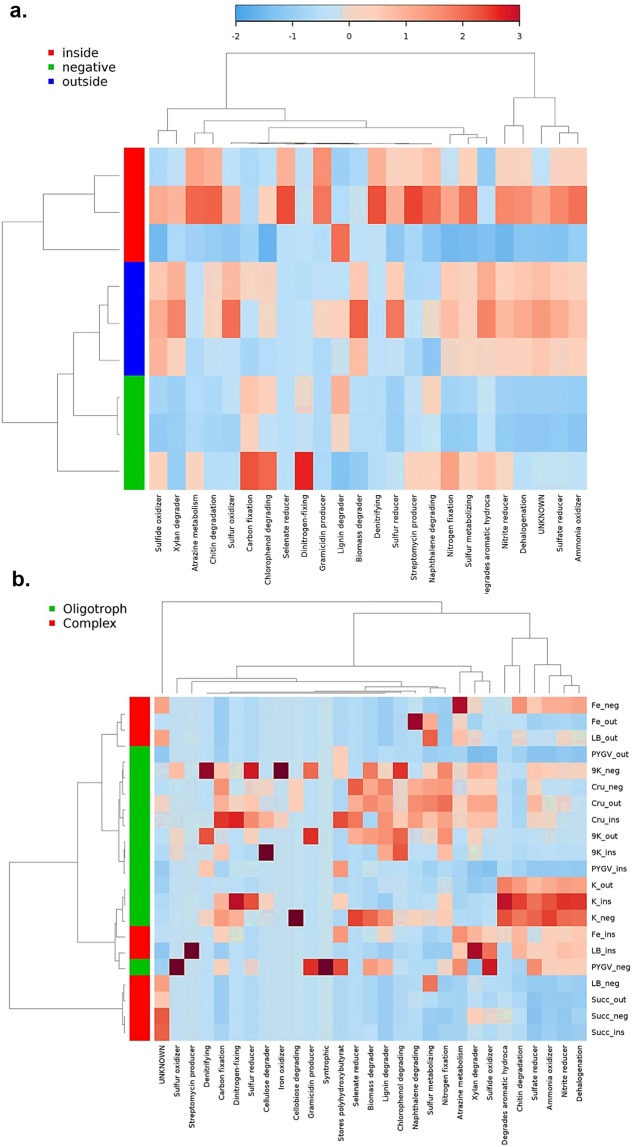
The metabolic profile of (**a**) environmental samples and (**b**) after the third round of enrichments from the Monte San Giorgio samples.

The taxonomic profile of the Monte San Giorgio microbiome depicts distinct communities related to the bituminous and non-bituminous samples which may possibly indicate distinct metabolic potentials (Fig. S1). To predict the metabolic capabilities of these microorganisms, their taxonomic profiles were fed to METAGENassist, which matches the taxonomic data with the phenotype databases^13^. The clustering analysis (heatmap) indicates the Monte San Giorgio shale microbiome is capable of degrading complex organics such as aromatic hydrocarbons, naphthalene, chlorophenol and atrazine; sulfate and nitrate metabolism is prominent in contrast to the limestone samples (Fig. 5a). The oligotrophic media in contrast to the nutrient-rich media have clearly enriched the shale microbiome with much diverse hydrocarbon metabolizing potential. For instance, 9 K, K, and crude oil minimal media enrich the microbes that can degrade chlorophenol, aromatic hydrocarbons, and naphthalene respectively.

#### Metagenomics data analysis

**Figure 6:**
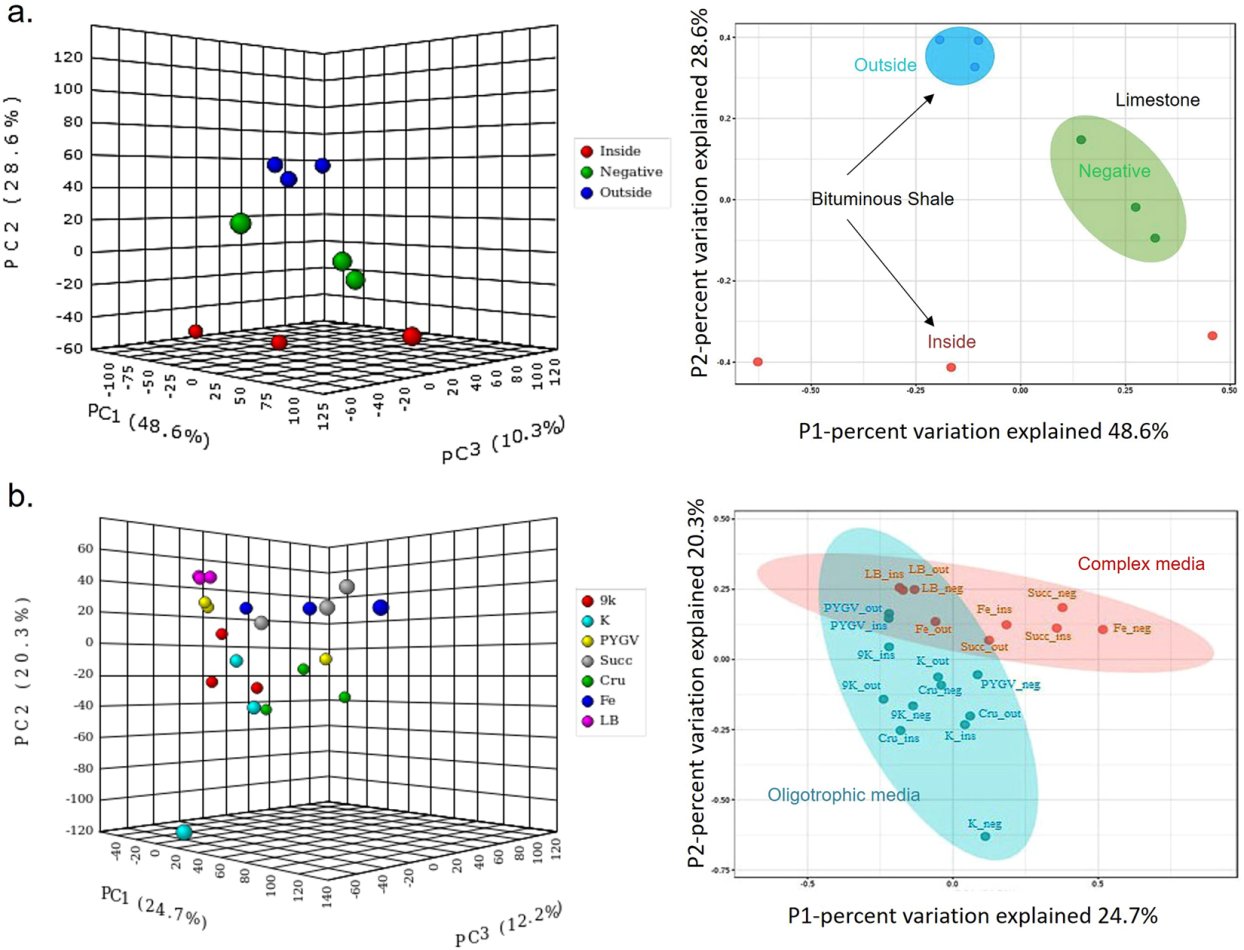
PCoA of the (**a**) Monte San Giorgio environmental samples and (**b**) after the third round of enrichments based on gene abundance data (K numbers). See Fig. 2 for abbreviations.

The abundance of functional genes was estimated from the 16s rRNA metagenomic data that allowed inference of possible function and mechanisms. Genome contents were predicted for each OTU and then the gene copy numbers were summarized by KO identifier (also called K number) retrieved from the KO database of functional orthologs (KEGG Kyoto Encyclopedia of Genes and Genomes Orthology)^21^. The PCoA based on these KO identifiers correlated with the PCoA predicted from the OTUs since some of the functional proficiencies (KO) are unique to the OTUs nearest matched genomes (Figs. 4 and 6). The functional profiles of the inside, outside, and negative limestone control samples differ from each other and the effect of the oligotrophic and complex media is also profound in selecting the microbes with the different functional profiles.

**Figure 7:**
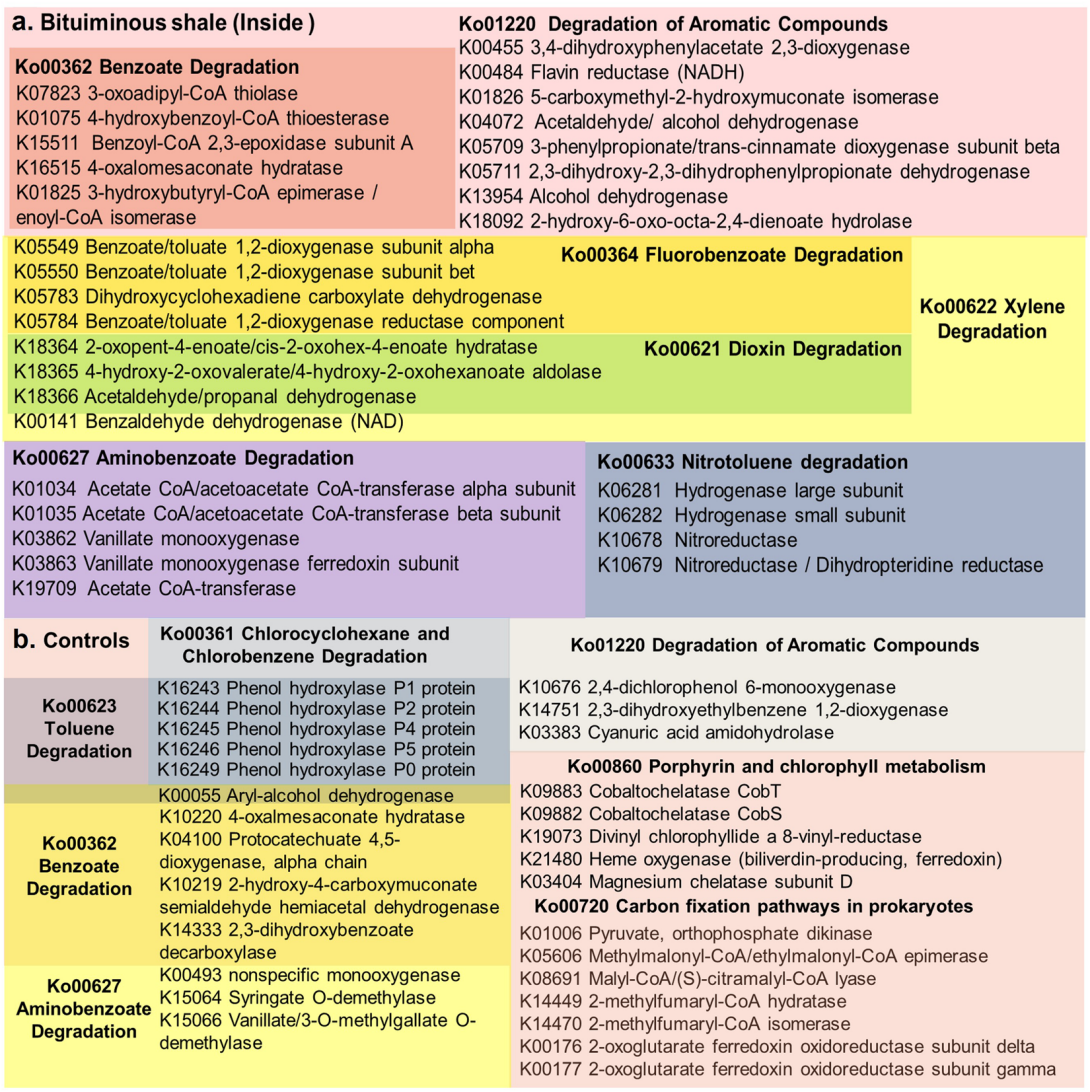
K numbers involved in the differentially abundant pathways (Ko numbers)^21^ of the bituminous shales (inside), against its outside surface and limestone samples (Controls). Enzymes shared by different pathways are shown as overlapping color boxes.

**Figure 8:**
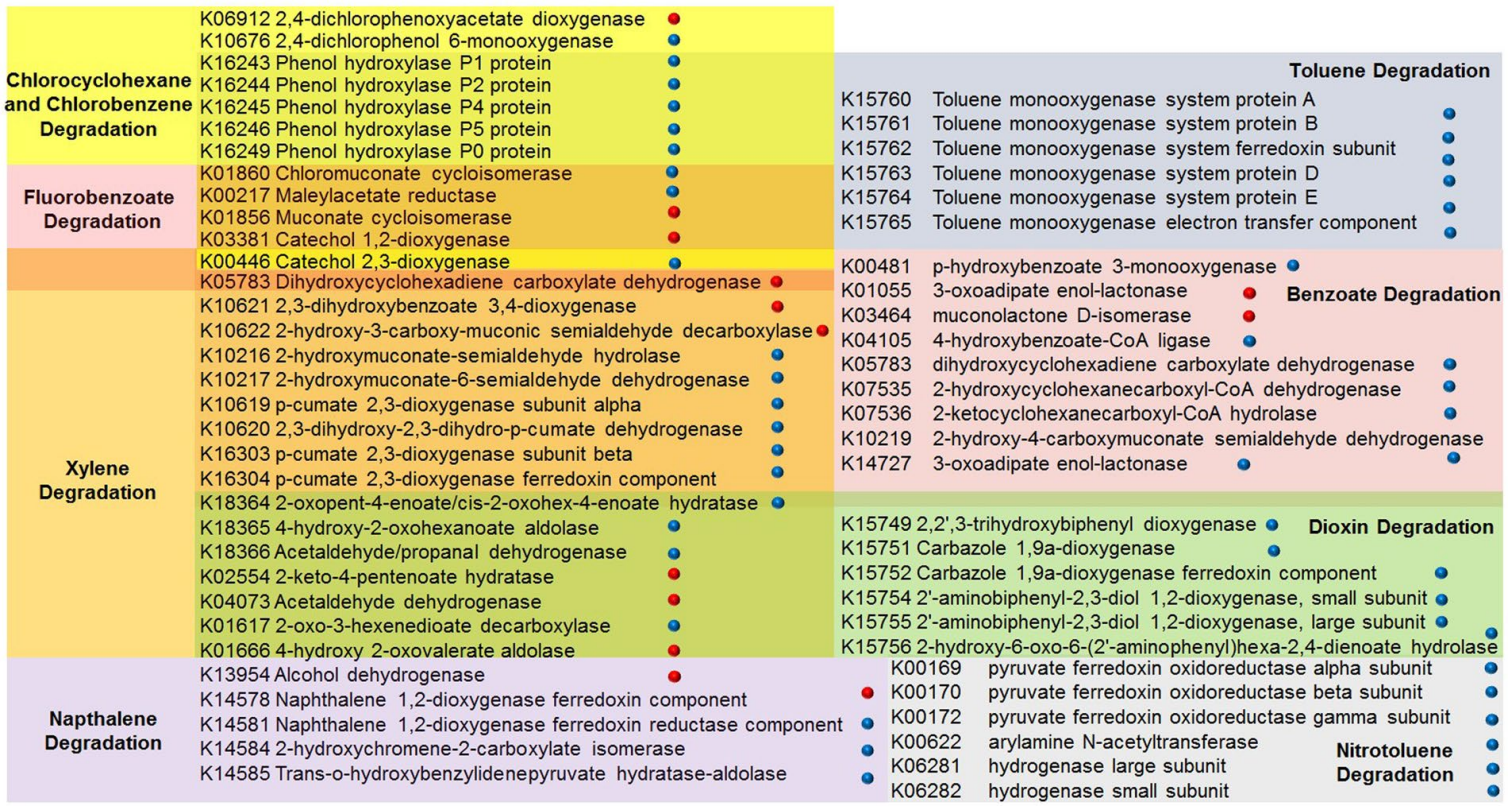
Differential abundance analysis of enriched microbes in the oligotrophic () and nutrient-rich media () after the third round of enrichments. Enzymes shared by different pathways are shown as overlapping color boxes.

To further elucidate the inferred key functional pathways in these biospecimen, differential abundance analysis for 16S rRNA Seq data was done with the statistical R packages DESeq2^22^. The gene abundance data retrieved from the FASTA sequences of the submitted 16s rRNA OTUs was summarized with KO abundances, KEGG reference database^21^. The significantly abundant features for bituminous shale (inside) against the outer surface of bituminous shale and limestone rock samples over 1-fold abundance difference were considered. The enzymes such as 4-hydroxybenzoyl-CoA thioesterase, benzaldehyde dehydrogenase, benzoate/toluate dioxygenase and vanillate monooxygenase, involved in the benzoate, xylene, fluorobenzoate, dioxin, aminobenzoate, and nitrotoluene degradation pathways were differentially abundant in the bituminous shale microbiome while the phenol hydroxylase, hydratase, 2 keto-4-pentenoate cobaltochelatase involved in the toluene, chlorocyclohexane, chlorobenzene, porphyrin, and chlorophyll metabolism were relatively abundant in the outside of the shale and limestone microbiome (Fig. 7). In order to know which enrichment media were more suitable for the selection and growth of microbes with bioremediation potential, the predicted key proteins and enzymes involved in the polycyclic aromatic hydrocarbon degradation pathways were analyzed in the oligotrophic and nutrient-rich complex media as described above. After the third round of enrichment, the oligotrophic media have selected microbes with key enzymes such as phenol hydroxylase, p-cumate 2,3-dioxygenase, toluene monooxygenase, p-hydroxybenzoate 3-monooxygenase, carbazole 1,9a-dioxygenase etc., potentially involved in the degradation of chlorocyclohexane, chlorobenzene, toluene, xylene, benzoate, naphthalene, and dioxin (Fig. 8).

## Discussion

About 95 million years ago, the progressive northwards movement of the African plate compressed the Eurasian plate, raising the seabed about 1,100 meters over sea level resulting in the emergence of the pyramid-like mountain, Monte San Giorgio. Most impressive is the fossil- and organic-rich series of Triassic and Jurassic sedimentary rocks (limestones, dolomites, marls, bituminous shales, etc.). Understanding its extant geomicrobiology would also expand our knowledge of shale bioweathering and long-term carbon cycling. With the 16s rRNA gene amplification and sequencing advances, the metagenomic structure and function of microbial communities inhabiting its bituminous shale and non-bituminous (limestone) rocks were investigated. The geomicrobiology of the Monte San Giorgio shale is similar to that of oil shale by-products - despite their geologically different origins - from Brazil (Irati Formation)^23^. The identified phyla *Acidobacteria, Actinobacteria, Bacteroidetes, Firmicutes, Nitrospirae, Proteobacteria, Chloroflexi, Cyanobacteria, Deinococcus-Thermus, Spirochaetes Gemmatimonadetes, Planctomyces*, and *Thermotogae* were also present in the black shale samples of Chengkou County, China, which covered the bottom zone toward the surface regolith^24^. However, bacteria involved in Fe-S cycling (*Acidithiobacillus, Sulfobacillus, Thiobacillus, Ferrimicrobium, and Ferrithrix)* and in anaerobic conversion of hydrocarbons to methane and CO_2_, catalyzed by syntrophic bacteria and methanogenic archaea, which are an important part of the dominant processes of black shale bioweathering, were absent in the Monte San Giorgio shale samples^15,24^.

Moreover, halotolerant *Proteobacteria* and *Firmicutes* (*Idiomarina*, *Marinobacter*, *Marinobacterium*, *Halomonas*, *Vibrio* and *Halanaerobium*) occurring in microbial mats from oil shale hydraulic fracturing wastewater treatment^25–27^ were also absent because the bioweathering of the Monte San Giorgio shale takes place under different topological and geochemical factors where desiccation stress at the exposed, terrestrial site and aerobic conditions should influence the microbial community. We suggest that the aerobic/facultative anaerobic orders (*Desulfurellales, Flavobacteriales, Rhizobiales, Xanthomonadales, Sphingobacteriales, Sphingomonadales, Caulobacterales, Bacillales, Lactobacillales, and Enterobacteriales*) and anaerobic orders (*Thermotogales, Thermoplasmatales, Desulfobacterales, Chromatiales, Desulfuromonadales*, *Hydrogenophilales, Campylobacterales*, *Rhodocyclales, Clostridiales*) found on the outside and inside surface of bituminous shale are responsible for the bioweathering and hydrocarbon degradation (Fig. S1)^15^. The majority of these orders belong to Proteobacteria. The sub-classes Alpha, Beta, Delta-, and Gammaproteobacteria have metabolisms capable of oxidizing a range of complex polymeric carbon compounds (including sugars, alcohols, organic acids, amino acids and carbohydrates) and complex hydrocarbons under aerobic and anaerobic conditions; underscoring the enhanced capacity for organic matter degradation^28–30^. The functional profiling predicted from the 16srRNA data also shed light on metabolic pathways with enzymes such as dioxygenases, thioesterases, hydrolases, aldoses, involved in the complex polycyclic hydrocarbon degradation that contributes to bioweathering of these bituminous shales. Of course, the validity of this assumption remains to be clarified and further studies are required to determine the underlying bioweathering mechanism.

In addition, we also studied how serial inoculum enrichment process enriches some microorganisms of the Monte San Giorgio shale communities over others. Choice of minimal media and extended incubation times facilitates the slow-growing, rarely isolated groups^31^. Complex media, such as LB, are preferred choice for the sake of convenience and their richness in carbon sources. Complex media allow the fast-growing copiotrophic microbes to consume nutrients and outgrow the slow growing chemoheterotrophic microbes, resulting in the substantial diversity loss. This general feature is also observed as a consequence of agricultural or other human activities, when increased input of nutrients in, e.g., water bodies, results in elevating the risk of eutrophication and reduced biodiversity^32,33^. Even just the nitrogen source addition can shift the bacterial diversity from oligotrophic towards a copiotrophic community^27,29,30,32,34,35^, but if the nutrients are limited, the fastidious microbes cannot simply outgrow the slow growing microbes, for instance in the crude oil medium, where the carbon source is not easily accessible. Thus, the oligotrophic media provide stringent conditions that keep a check on the growth of fast-growing microbes but let the slow-growing microbes to grow and proliferate. Consequently, the diversity loss in the oligotrophic media is not as drastic as in the complex media. Additionally, oligotrophic media are more suitable for the selection and growth of slow-growing specialized microbes in bioremediation and polycyclic aromatic hydrocarbon degradation that also differentially expressed key enzymes such as phenol hydroxylase, p-cumate 2,3-dioxygenase, toluene monooxygenase, p-hydroxybenzoate 3-monooxygenase, carbazole 1,9a-dioxygenase involved in the chlorocyclohexane, chlorobenzene, toluene, xylene, benzoate, nitrotoluene, naphthalene, and dioxin degradation.

## Conclusion

Our study provides the first insight into modern microbial diversity and metabolic types of the bituminous shale and limestone rocks from Monte San Giorgio. It explores how different environmental conditions (organic-rich shale and nutrient-deprived limestone) aid in selection and habituation of the different microbiomes from the environment (soil contaminated outer surface of shales). Moreover, this study expands our understanding about serial subculturing of the microbial communities in general, which will improve our choice of media for enriching microbes with biotechnological and bioremediation potential. The computational tools anticipate further that the Monte San Giorgio shale microbiome has specialized metabolic pathways involved in the complex hydrocarbon degradation and the oligotrophic media are preferred for enriching such microorganisms with particular biochemical and bioremediation applications.

## Material and Methods

### Sampling site

Rock samples were collected in Jul-2017 from the site Acqua del Ghiffo (45°54′20.4″N 8°55′54.4″E), situated at Monte San Giorgio (Fig. S3). Samples were taken from an outcropping bedrock with a rock hammer and chisel and immediately wrapped in sterile aluminium foil until further use. The MSG-17-1 samples were organic-rich bituminous shale derived from the Cava superiore and Cava inferiore strata while MSG-17-2 were derived from non-bituminous Meride Limestone and used as the negative control for this study. Samples were not directly touched, in order to avoid contamination with organisms adhering to skin and clothes. If necessary, tools were treated with ethanol and cleaned with a sterile cloth prior to use. Care was taken that the sterilizing liquid was completely evaporated before sampling. The sampling sites, as well as the samples, did not have direct contact with a soil cover, in order to avoid direct contamination with soil particles and leaf litter. Rock masses of 700–1200 g were collected, in order to reduce desiccation of subsamples to be taken just prior to DNA extraction. Samples were kept wrapped at ambient temperature until further analysis. At least three samples of the MSG-17-1 and MSG-17-2 were used for the DNA extraction. Samples for DNA extraction (0.4 g each) were taken by scratching the outer surface of a rock piece (outside sample) with a sterile scalpel in the vicinity of a bunsen burner. The slabby rock was then carefully cleaved and the freshly exposed surface (exhibiting a characteristic diesel smell) was again sampled with a sterile scalpel (inside sample).

### DNA extraction and Illumina sequencing of the Monte San Giorgio samples

The genomic DNA of the microbial community from inside and outside samples of the MSG-17-1 rock samples was extracted by the Powersoil DNA isolation kit (Qiagen, Venlo, The Netherlands) according to the manufacturer’s instructions. For each sample, 0.4 g of scratched material was used to extract soil microbial genomic DNA while blanks were also assessed for each sample to eliminate possible reagents contamination with microbial DNA. Essentially, in DNA isolation with this kit, the total genomic DNA was captured on a silica membrane in a spin column after inhibitors removal and protein precipitation. The same procedure was performed for the limestone rocks which served as the negative control. Followed by washing and elution, the quality and quantity of the extracted genomic DNA were checked with gel electrophoresis using 0.8% agarose in Tris-acetate-EDTA (TAE) buffer, pH (8.3)^36^ and photometrically in a Nanodrop ND-1000 spectrophotometer (PeqLab, Germany), respectively. No DNA contamination was observed in the blanks after the DNA extraction and the subsequent PCR amplification. The variable regions V3-V4 of the 16s rRNA gene was amplified with the Illumina overhang adapters on their 5′ end of the 16S Amplicon Miseq PCR Forward 5′-TCGTCGGCAGCGTCAGATGTGTATAAGAGACAGCCTACGGGNGGCWGCAG-3′ and Reverse 5′-GTCTCGTGGGCTCGGAGATGTGTATAAGAGACAGGACTACHVGGGTATCTAATCC-3′ primers^37^ with the PCR profile with some modifications^38^. A GeneRead Size Selection Kit (Qiagen, Germany) was used to remove primers and dimers from the resulting amplicons. Indexing of these PCR products was performed with Nextera XT DNA library prep kit (Illumina, San Diego, Cal, USA) according to manufacturer’s instructions. Paired-end sequencing was performed in collaboration with Göttingen Genomics Laboratory with an Illumina MiSeq sequencer (Illumina). Blanks were automatically discarded during the processing of sequences due to very low read counts.

### Enrichment media on microbial communities

The inoculum from the inside, outside, and negative MSG samples were enriched in different media (9 K^39,40^, K^41^, PYGV^42^, succinate minimal media^43^, minimal media supplemented with 1% crude oil^44^, iron basal media, and LB media^45^) (Table S3). The culture media were re-inoculated after every week and after 3 cycles of inoculum subculture and incubation at room temperature with rotary shaking at 120 rounds per minute, a subset of microbes promoted by the specific growth medium is supposed to be fully enriched. The genomic DNA was extracted from the enrichments after 1st and 3rd round of incubation with the Powersoil DNA isolation kit (MoBio Laboratories) subjected to DNA extraction and sequencing as described above.

### Metagenomic analysis via MetaAmp pipeline

The Illumina amplicon sequencing data was fed to the online available MetaAmp automated pipeline for metagenomic analysis (http://ebg.ucalgary.ca/metaamp/)^46^. First, usearch -fastq_mergepairs assembled the demultiplexed and uncompressed fastq format sequence files as paired-end reads via USEARCH software package^47^. The read pairs shorter than 350 bp length, misaligned and mismatched in the overlap region were removed. Next, the forward and reverse primers were identified and trimmed in the Mothur software package via trim.seqs command^48^. The reads without the forward and reverse primers or with mismatches in the primer region were discarded. In the quality filtering step, the low-quality reads were removed to minimize the influence of sequencing errors using usearch -fastq_filter command with -fastq_trunclen -fastq_maxee -fastaout options in USEARCH. The high-quality reads were labeled with the unique sample ids and the reads from different files are pooled together. Next, the UPARSE software^49^ was used to dereplicate reads, to discard singletons and chimaeras, and to cluster the pooled high-quality reads into operational taxonomic units (OTUs). The OTU clustering threshold is 0.97 that is 97% identity. The OTUs were assigned taxonomic status with the classify.seqs command in Mothur that utilize the reference SILVA training dataset (http://www.mothur.org/wiki/Taxonomy_outline). In the end, Mothur generated rank-abundance data, rarefaction curves, alpha-diversity indexes and beta-diversity, followed by rarefying samples into subsamples. In addition, principal coordinate analysis (PCoA) was also calculated using the Bray-Curtis index to compute dissimilarities among different samples. Hypothesis testing was done with the unifrac.weighted^19^ and Analysis of Molecular Variance (AMOVA)^20^ tests.

### Functional profiling

The taxonomic profile data obtained from the MetaAmp was further processed by the METAGENassist online Server to give an overview of the functional profiles of different samples^13^. The comparative metagenomic analysis was performed with the clustering algorithm ward in relation to interpret the metabolic profiles of the predicted microbiomes. More in-depth details of the enzymes and metabolic pathways involved were obtained from another recently developed online tool Piphillin (http://secondgenome.com/Piphillin)^14^ which directly searches each representative FASTA sequence of the submitted OTUs against USEARCH version 8.0.1623^47^. Gene copy numbers for each genome were summarized with KO abundances, KEGG reference database^21^. The comprehensive statistical and meta-analysis including differential abundance analysis of gene abundance data was completed with the online tool MicrobiomeAnalyst^50^ and its R packages DESeq2^22^. The significant features for bituminous shale (inside) against the outer surface of bituminous shale and limestone rock samples over 1-fold difference were considered to summarize the results.

## Supplementary information

Supplementary info
