# Supplementary material for: Composition, Diversity and Functional Analysis of the Modern Microbiome of the Middle Triassic Cava Superiore Beds (Monte San Giorgio, Switzerland)
Source: Sci Rep. 2019 Dec 31;9:20394. doi: 10.1038/s41598-019-55955-5 (PMC6938485; doi:10.1038/s41598-019-55955-5)
Supplement: Supplementary file 1 — Supplementary info [file 41598_2019_55955_MOESM1_ESM.docx]

## Supplementary information

**Composition, Diversity and Functional Analysis of the modern Microbiome of the middle Triassic Cava Superiore Beds (Monte San Giorgio, Switzerland)**

Sania Arif^1*^, Joachim Reitner^2^, Michael Hoppert^1^,

^1^ Institute of Microbiology & Genetics, Department of General Microbiology; ^2^Geosciences Centre, Department of Geobiology; Georg-August-Universität Göttingen

*Correspondence to sania.arif@stud.uni-goettingen.de

**Figure S1, Figure S2, Figure S3, Table S1, Table S2, Table S3**

*Supplementary Information*


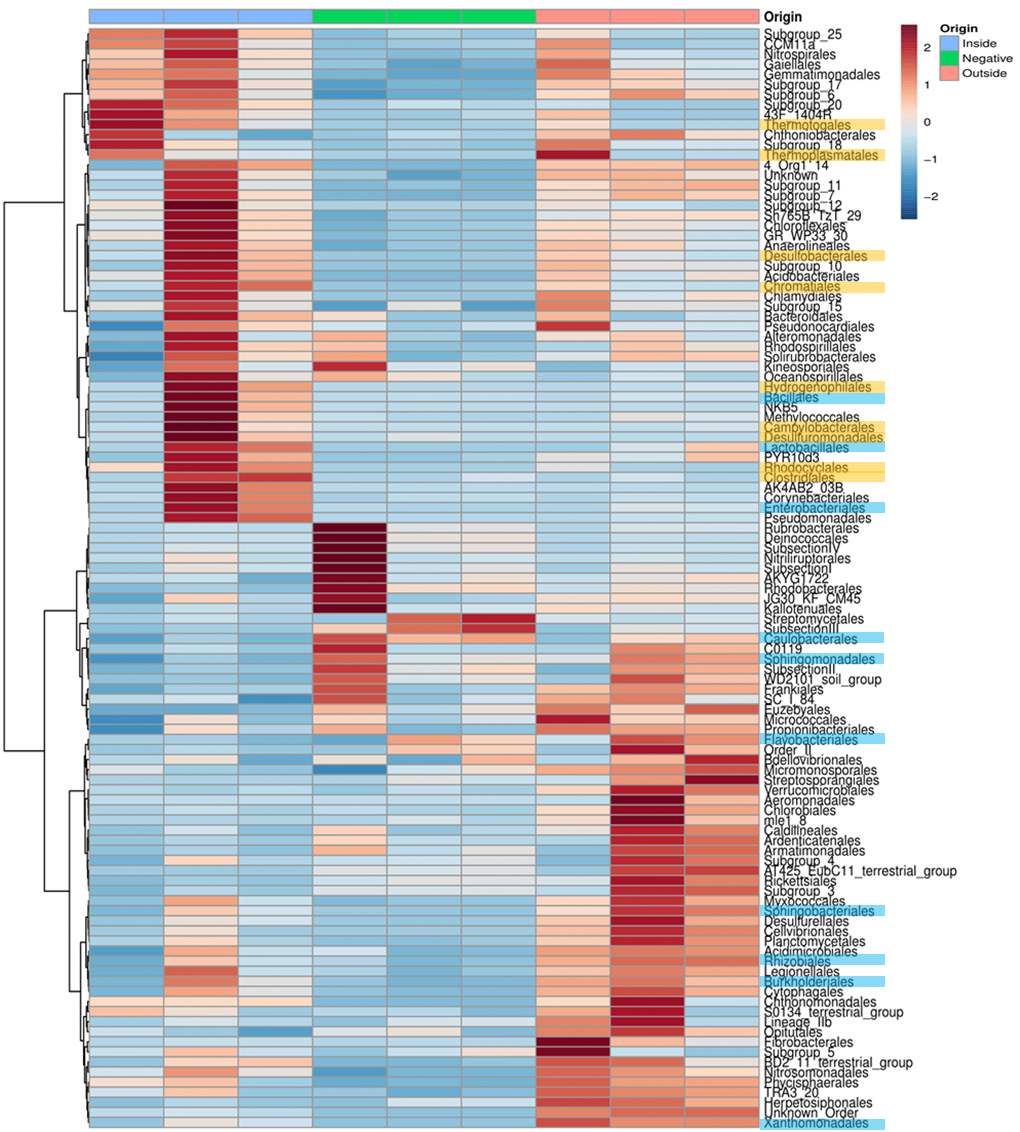


**Figure S1.** Heatmap of the Monte San Giorgio geomicrobiology at the order level. The known already reported anaerobic and the aerobic/facultative orders involved in the hydrocarbon metabolism are highlighted in yellow and blue respectively.


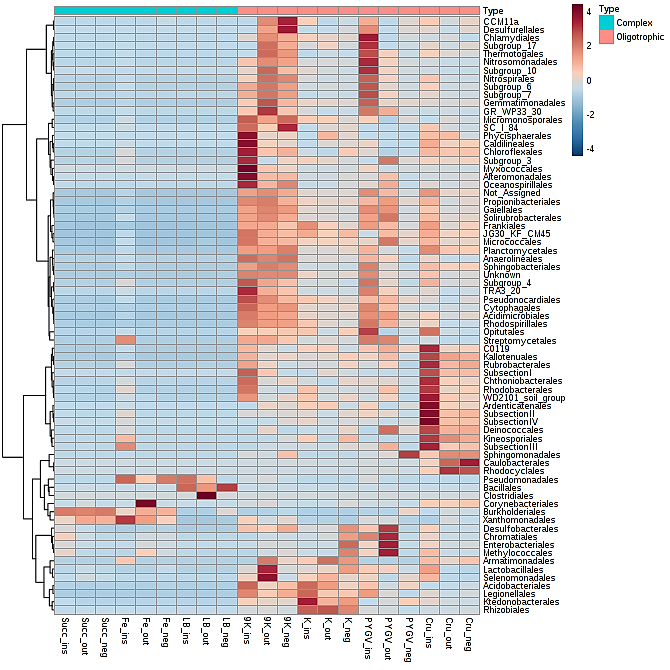


**Figure S2**. Heatmap of the orders, enriched in the nutrient-rich complex and oligotrophic media from Monte San Giorgio rock samples, after third and final round of enrichment.


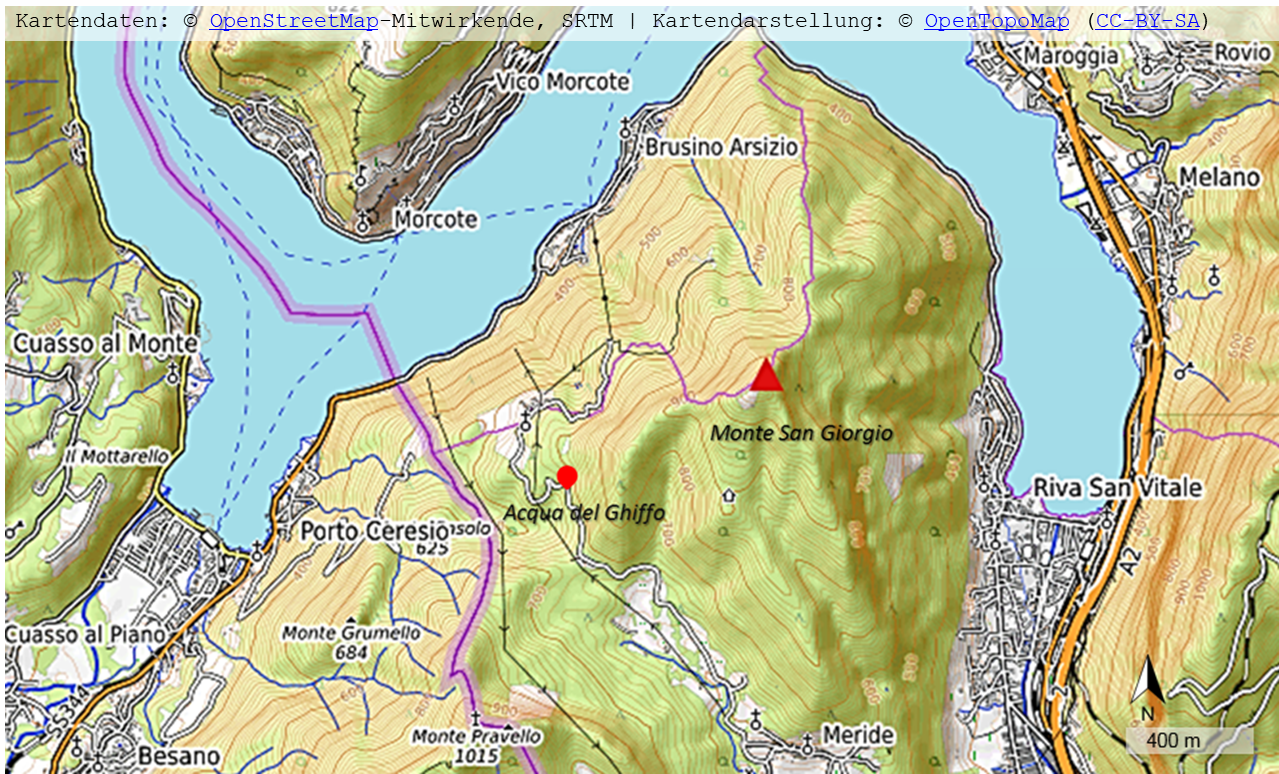


**Figure S3.** Map of sampling site (Acqua del Ghiffo) in the Monte San Giorgio mountain range. The map was reproduced from OpenTopoMap (https://opentopomap.org), under creative common license CC BY-SA 3.0 (https://creativecommons.org/licenses/by-sa/3.0/legalcode), subsequently marked with the sampling site and area.

**Table S1.** Alpha diversity indexes of the Monte San Giorgio environmental samples

|  | Observed OTUs | Chao1 | ACE | Shannon | Simpson |
| --- | --- | --- | --- | --- | --- |
| Outside | 2712+141.5 | 3105+55 | 3175+49 | 6.5+0.005 | 0.004995 +0.000087 |
| Inside | 2012+65 | 2522+18 | 2575+16 | 6.1+0.005 | 0.0071915+ 0.0001615 |
| Negative | 1991+18 | 2440+65 | 2483+62 | 6.04+0.017 | 0.007062+0.000346 |

**Table S2.** Analysis of molecular variance and unifrac analysis of the Monte San Giorgio environmental samples

| **Analysis of Molecular Variance (AMOVA)** |
| --- |
| C-P-T Among Within Total  SS 0.719341 0.853931 1.57327  df 2 6 8  MS 0.359671 0.142322  Fs: 2.52716  p-value: 0.005*  C-P Among Within Total  SS 0.378582 0.537901 0.916483  df 1 4 5  MS 0.378582 0.134475  Fs: 2.81526  p-value: 0.099  C-T Among Within Total  SS 0.445195 0.636744 1.08194  df 1 4 5  MS 0.445195 0.159186  Fs: 2.7967  p-value: 0.101  P-T Among Within Total  SS 0.255234 0.533217 0.788451  df 1 4 5  MS 0.255234 0.133304  Fs: 1.91468  p-value: 0.086 |
| **Unifrac.weighted** |
| Tree# Groups WScore WSig  1 C-P 1.000000 <0.0010  1 C-T 1.000000 0.0190  1 P-T 0.889840 <0.0010 |
| **C** Negative control **T** Outside **P** Inside |

**Table S3.** Details of Enrichment Media

| Medium | Components per litre distil H_2_O | Features | Growth | References |
| --- | --- | --- | --- | --- |
| 9K | (NH_4_)_2_SO_4,_ KCl, K_2_HPO_4,_ MgSO_4_⋅7H_2_O  Ca(NO_3_) _2,_ FeSO_4_⋅7H_2_O  H_2_SO_4_ (1N) | Acidic medium | Iron-oxidizing bacteria | Silverman, M.P. & Lundgren, D.G. 1959 ^35^  Yates, J.R. & Holmes, D.S. 1987 ^36^ |
| K | MnSO_4_⋅H_2_O, FeSO_4_⋅7H_2_O, peptone, yeast extract, and 10 mM HEPES buffer, pH 7.5 | Manganese containing complex media | Heterotrophic Mn oxidizers | Rosson, R.A., Tebo, B.M. & Nealson, K.H. 1984 ^37^ |
| PYGV | Peptone, Yeast Extract, Mineral Salt and Vitamin Solution | Low-nutrient medium | Slow growing and sheathed bacteria | Ghiorse, W.C. & Hirsch, P. 1982 ^38^ |
| Succinate Minimal media | Succinate, FeSO_4_, MnCl_2_, NHCl_,_  KCl, K_2_HPO_4_, Hepes Buffer, Vitamin Solution | Basal minimal medium | Mn binding and metabolism | Kepkay, P.E. & Nealson, K.H. 1987 ^39^ |
| Fe basal media | Na_2_HPO_4_⋅12H_2_O, KH_2_PO_4,_ NH_4_Cl, MgSO_4_⋅7H_2_O, CaCl_2_⋅2H_2_O, Fe(III)-NH_4_-Citrate, SL-10 Trace Elements Solution | Sodium acetate as carbon source | Fe(III)-NH_4_-Citrtate as electron acceptor | Customized for this study |
| Crude oil with Minimal Media | MgSO_4_, CaCl_2_, KH_2_PO_4_, K_2_HPO_4_, NH_4_NO_3_, and FeCl_3_, 1% crude oil and pH adjusted to 7-7.2 | Complex organics containing media | Crude oil degrading Bacteria | Liu, Z., Jacobson, A.M. & Luthy, R.G. 1995 ^40^ |
| Luria-Bertani LB medium | Tryptone, yeast extract and NaCl | Nutritionally-rich medium | Fast growing bacteria, fastidious protein degraders | Bertani, G. 1951 ^41^ |
